# Oligometastatic adrenocortical carcinoma: definition and treatment

**DOI:** 10.1097/CCO.0000000000001209

**Published:** 2025-11-25

**Authors:** Marta Laganà, Alfredo Berruti, Salvatore Grisanti, Deborah Cosentini

**Affiliations:** Department of Medical and Surgical Specialties, Radiological Sciences, and Public Health, Medical Oncology, University of Brescia at ASST Spedali Civili, Brescia, Italy

**Keywords:** adrenocortical carcinoma, locoregional therapy, mitotane, multimodal management oligometastatic adrenocortical carcinoma can be reasonably defined as stage IVa or ≤5 metastases <3 cm, oigometastatic disease

## Abstract

**Purpose of review:**

Oligometastatic adrenocortical carcinoma (ACC) represents a distinct clinical subset of metastatic disease characterized by a limited tumor burden and potentially indolent biology. This review summarizes current evidence on its definition and management strategies.

**Recent findings:**

Although mitotane and EDP-M chemotherapy remain the backbone of systemic therapy for advanced ACC, increasing evidence supports integrating local treatments – such as surgery, stereotactic body radiotherapy (SBRT), image-guided thermal ablation (IGT), and transarterial embolization (TACE/TARE) – in selected patients. Retrospective studies suggest that individuals with ≤5 metastases or lesions <3 cm, often classified as stage IVa, achieve higher disease control rates and prolonged survival when local and systemic therapies are combined. Decision-making should consider patient fitness, tumor biology (Ki-67 index, time to recurrence), and prior treatments within a multidisciplinary framework.

**Summary:**

If a definition of oligometastatic ACC is required, a reasonable one would include stage IVa disease or up to five metastases <3 cm. Management should rely on a multidisciplinary approach in referral centers, integrating systemic and local therapies to optimize survival and quality of life.

## INTRODUCTION

Adrenocortical carcinoma (ACC) is a rare endocrine malignancy, potentially highly aggressive, often diagnosed at advanced stage. Surgery remains the gold standard for resectable adrenocortical carcinoma (ACC), whereas first-line treatment for metastatic disease is based on polychemotherapy with the EDP-M regimen [1,2].

Oligometastatic disease (OMD) is typically defined as the presence of a limited number (commonly 1–5) of metastatic lesions, confined to a few organs [3], with the assumption that the metastatic potential of the cancer is restricted and potentially amenable to curative-intent local therapies in selected cases. In ACC, defining oligometastatic disease is particularly challenging due to: potential aggressive biology and heterogeneity of disease course/survival, frequent presentation with widespread disease at diagnosis, lack of consensus on whether “limited metastases” reflect a true biologically distinct state.

Roux *et al.* proposes the definition of oligometastatic ACC as stage Iva (≤2 metastatic site) patients with 5 metastases or a maximum metastasis diameter below 3 cm basing on better disease control rate with local therapy in this subgroup of patients [4^▪^].

The first challenge lies in deciding whether to initiate a standard first-line regime, such as EDP-M chemotherapy, in patients with oligometastatic ACC, either de novo or at recurrence after surgery. This decision must also take into account the potential role of local treatments, both on the primary tumor and/or on metastatic sites, in order to better control the progression disease and/or postpone the systemic therapy.

Second and later lines approaches are not standardized, as no prospective trials have consistently demonstrated important responses [1]. Consequently, therapeutic decisions are highly variable and depend on factors such as progression-free interval after first-line therapy, tumor burden, prior toxicities, comorbidities, and drug availability or reimbursement. Also in this context, the use of local treatments may represent a reasonable option to maximize the disease control; however, their integration into clinical management is challenging and should be considered on a case-by-case basis within a multidisciplinary setting.

In this review, we aim to summarize and critically interpret the available evidence regarding management of oligometastatic ACC. 

**Box 1:**
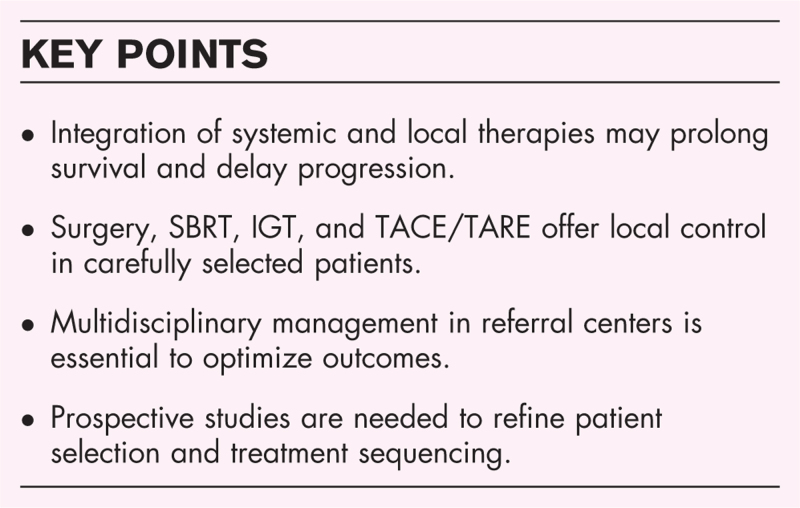
no caption available

## OLIGOMETASTATIC ADRENOCORTICAL CARCINOMA APPROACH

In the setting of oligometastatic ACC, treatment strategies are highly individualized and must take into account multiple clinical and biological factors.

Therapeutic options in this scenario may therefore include mitotane monotherapy, polychemotherapy in combination with mitotane, locoregional treatments, or a combined approach integrating local and systemic therapy.

**FIGURE 1:**
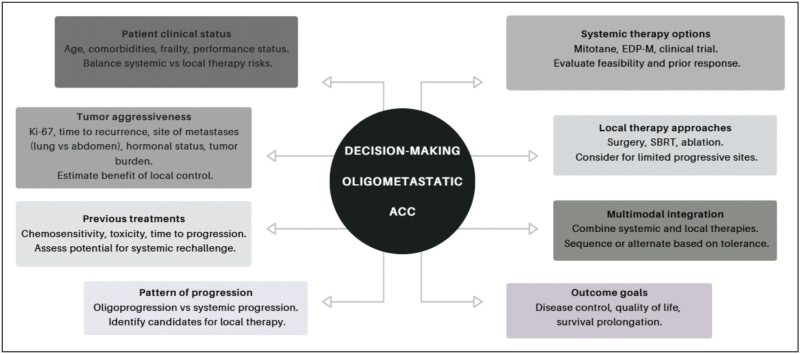
Decision making key points in oligometastatic disease.

The choice of therapy may depend on the site of metastatic involvement and the functional status of the tumor, as hormonally active lesions may require specific and timely interventions. Equally relevant are the number of metastatic lesions and the overall tumor burden, which influence both the feasibility and the expected benefit of local approaches. The aggressiveness of the disease is reflected not only by histopathological features such as the Ki-67 index but also by the clinical course, including the interval to recurrence or progression after initial therapy (Fig. 1).

Local therapy in oligometastatic ACC has to be considered in the following situations:

(1) Lesion/s progressing or not responding to systemic therapy in the context of indolent or slowly progressive disease.
(2) To postpone systemic polychemotherapy in frail patients.
(3) Appearance of new lesions after a long recurrence or progression-free interval.

## LOCAL TREATMENTS: LITERATURE SUPPORT

Current guidelines^1^ recommend surgery or alternatively other local therapies in patients with recurrent disease and a disease free interval of at least 12 months (or an indolent disease course or under therapeutic control in whom systemic therapy led to an objective response or long-term stable disease), in whom a complete resection/ablation seems feasible [5–7].

Imaging-guided locoregional treatments (IGTs) include a range of procedures to achieve local tumor control. These procedures include image-guided thermal ablations (such as radiofrequency ablation, microwave ablation, and cryoablation) and trans-arterial embolization (such as chemoembolization and radio-embolization). IGTs are commonly used to achieve local control of many malignancies, but reported experience with IGTs in ACC is limited [8].

A recent review included 21 retrospective papers and reported on 374 patients treated with local therapy for advanced ACC (12 studies on radiotherapy, 3 on transarterial chemoembolization and radioembolization, 4 on image-guided thermal ablation [radiofrequency, micro wave ablation, and cryoablation, and two studies reporting treatment with several different LT]). Radiotherapy was frequently performed with palliative intention. Patients with a disease-free interval after primary surgery of more than 9 months and lesions <5 cm might benefit most. Local thermal ablation was the most common technique (84 patients) and liver and lung the most common treated sites [9^▪^].

In a retrospective cohort treated at the MDAnderson cancer center IGTs showed a disease control rate of (70 out of 86 lesions treated) having low risk for severe complications [10]. At baseline, 15 out of 26 patients (57.7%) had hormonally active disease, 9 (34.6%) were nonfunctional. Twenty-two of the 26 patients (84.6%) had previously undergone surgery on the primary tumor. Prior systemic therapy included mitotane in 13 patients (50.0%), (most chemotherapy and immunotherapy). A total of 51 image-guided thermal (IGT) procedures for 86 lesions were performed, with a median of 2 lesions ablated per patient (range 1–9). The median number of IGT procedures per patient was 1 (range 1–4). Cryoablation was the most frequently used technique, performed in 49 procedures (57.0%). The majority of lesions were localized to the liver (37, 43.0%) or intraperitoneal sites (11, 12.8%) and median dimension of treated lesions was 2.3 cm (range 0.5–8.6 cm). Concurrent systemic therapy at the time of the first IGT procedure consisted of mitotane in six patients (23.1%), immunotherapy in five (19.2%), and chemotherapy in three (11.5%). Overall, 61.5% of patients subsequently received systemic therapy during follow-up, most commonly chemotherapy or immunotherapy. The median time to the next line of systemic therapy was 12.5 months. Adverse events related to IGT procedures were infrequent and predominantly low grade. Specifically, two cases of postprocedural hematoma, one pneumothorax, and one biloma were observed. No treatment-related mortality occurred. The estimated 2-year overall survival rate was 72%.

A retrospective study evaluated stage IVa (≤2 metastatic sites) ACC patients treated in a referral center with mitotane and locoregional therapy (LR) included 60 patients and 109 local therapy procedures. Disease control rates were higher in patients with ≤5 metastases or a maximum metastasis diameter <3 cm. Thirty-five percentage of patients experienced a combination of different type of loco-regional treatments, liver and lung were the most frequent sites (36 and 28 patients, respectively). Chemoembolization and radiofrequency were the most common procedures and median overall survival (OS) was 68 months.^4^

Boileve *et al.* support the mitotane plus locoregional strategy in a well defined population of 79 ACC stage IVA. A favorable impact on progression and long survival (around 4 years) was observed and 13% of CRs are reported. Most frequent approaches were surgery for local relapse (23 patients), TACE (20 patients) and Radiofrequency (18 patients) for liver and lung metastases [11].

Mauda-Havakuk *et al.* also reported in 2021 that the use of a combination of IGTs, chemotherapy, and surgery in 39 patients with advanced ACC was associated with longer 2- and 5-year survival in comparison to the use of surgery and chemotherapy alone or chemotherapy and radiation therapy alone. Liver and lung were the most common treated metastatic sites [12].

## LOCAL THERAPY OPTIONS

The most critical part in applying local therapy to patients with ACC is probably the selection of the most adequate method to the “right” patient and this choice depends heavily also on local experience and expertise. For some localizations certain methods seem to be more suit able than others (e.g., RT for bone or cerebral metastases) and for some modalities the cumulative experience in certain organs is much higher than in other parts of the body (e.g., TACE for liver lesions).

### Radiotherapy

Classically, ACC was considered a radioresistant disease. This assumption was based on results from very small series in which radio therapy (RT) was used for palliation. More recent studies report improved tumor control being achieved by delivering higher doses using more accurate techniques offering potential for local control in oligometastatic disease. More experience in an adjuvant setting are available and showed to reduce the risk of local recurrence.

In a recent review 200 patients with advanced ACC treated with RT were described, in 13 retrospective studies with very heterogeneous and not-conclusive results [13]. However, considering the largest study, including 132 irradiated tumor lesions with only 14 (11%) showing progression, while all other lesions reaching stable disease or objective tumor response in 89%. In the subgroup of patients with conventional RT with a higher dos age (50–60 Gy) or with stereotactic body radiotherapy the objective response rate was even 95 and 100%, respectively. Bone, lung and local recurrence were the most common sites irradiated.

Only few studies investigated possible predictive factors influencing response to RT, identifying the absence of glucocorticoid excess and a Ki67 ≤15% as possible predictive factors. Roux *et al.* could show that a maximum of five metastases or a maximum diameter below 3 cm were associated with higher rates of disease control (however, this result refers to all locoregional treatments collectively, and not specifically to radiotherapy).^4^

### Thermal ablation

Radiofrequency ablation, microwave ablation and cryoablation are minimally invasive percutaneous thermal ablation therapies and were collected in 6 studies covered 102 patients with ACC treated with at least one or more image-guided thermal ablation therapy.

Considering cryoablation, liver and intraperitoneal lesions, (median dimension 2 centimeters), were the most used at the MDAnderson cancer center retrospective study.^10^

A total of 132 tumoral lesions (liver lesion in the 60% of cases, median dimension of 2 cm) were collected in a multicenter study and 66 patients were treated with local thermal ablation (*n* = 84) and complete response was achieved in 27 lesions (20.5%; all of them achieved by LTA). Time to progression was particularly long in patients treated with LTA (median not yet reached).^9^

### Transarterial chemo embolization

Data on transarterial chemo embolization (TACE) as treatment for patients with advanced ACC are very limited and retrospective, no high grade adverse events were reported.

In the largest series of patients (*n* = 29) treated with TACE in advanced ACC a decrease in tumor size in 22% of 103 treated lesions was observed, an additional 65% of lesions were stable in size after 3 months, interestingly higher response rates were observed in lesions with a diameter <3 cm [14].

Roux *et al.* reported a significantly higher rate of disease control in a subgroup of cases (≤5 metastases or maximum diameter < 3 cm), 20 patients out of 60 were treated with TACE. Mauda-Havakuk *et al.* stated a possible prolonged life expectancy in patients (*n* = 39) after loco-regional treatment (TACE: *n* = 12).^4^ The use of transarterial radioembolization (TARE) was described in three patients with ACC and liver metastases showing a longer overall survival in comparison to patients without TARE (32.4 months vs. 9.9 months, *P* = 0.011) [15]. Considering the cohort study including 132 lesions, TA(C)E (*n* = 40), and TARE (*n* = 8), time to progression of the treated lesion was only 8.3 months after TA(C)E and 8.2 months after TARE [9^▪^].

### Surgery

Surgery should be performed only by surgeons experienced in adrenal and oncological surgery aiming at a complete en bloc resection of the ACC or the metastases. A cytoreductive resection may also be indicated in rare cases of severe symptomatic hormone excess, after attempts to control the symptoms with a combination of fast-acting antisecretory agents (i.e. metyrapone) and mitotane and local therapies. In these patients, postoperative mitotane is clearly advised [16].

Retrospective data suggest that resection of primary tumor should not be overlooked in patients with metastatic ACC if deemed safe and feasible [17]. Nevertheless, we support the cytoreductive chemotherapy that can help in selecting candidates for surgery and may reduce the need for extensive resection in those who respond well to systemic therapy. In a phase II trial, 9 patients underwent cytoreduction and HIPEC and in a retrospective monocentric study 14 relapsed ACC were treated with results encouraging the investigation of this strategy [18].

### Clinical case: integration of treatments with durable disease control of oligometastatic adrenocortical carcinoma patient

A 52-year-old woman, with no significant comorbidities, was diagnosed in 2016 with adrenocortical carcinoma (ACC) with pulmonary metastases (T3N0M1, Ki-67 10% deriving from adrenal mass biopsy) following evaluation for secondary amenorrhea. She was sent to our center and received six cycles of first-line EDP-M chemotherapy, achieving a partial response on all disease sites. In 2017, she underwent right adrenalectomy (Ki-67 3–5%) and surgical resection of two pulmonary nodules, both consistent with metastatic ACC. Adjuvant mitotane was continued within therapeutic range.

In 2021, pulmonary progression led to enrollment in a clinical trial with immune checkpoint inhibitors, obtaining a partial response of bilateral lung metastases. In 2022, following progression of a single 6-cm lesion extending from the retrocaval area to the right atrium, she was withdrawn from the study and underwent surgical resection (Ki-67 15%). Mitotane therapy was resumed.

**FIGURE 2:**
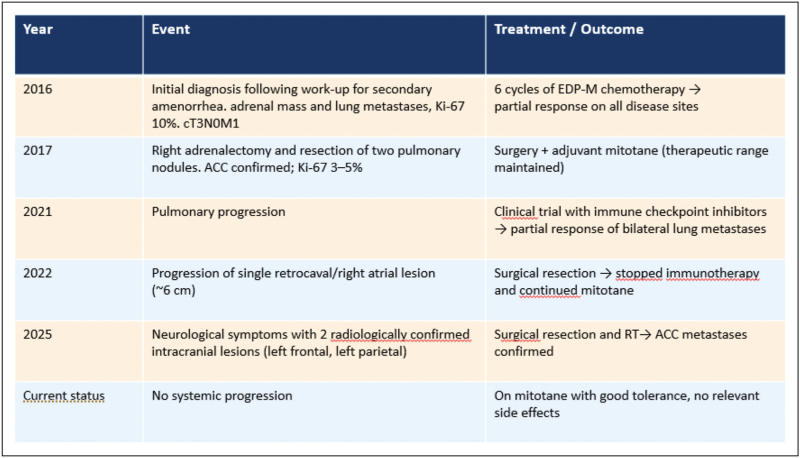
Clinical timeline of a 52 years old woman with oligometastatic ACC. ACC, adrenocortical carcinoma.

In March 2025, due to neurological symptoms, brain CT revealed two lesions (left frontal and left parietal), both surgically removed and confirmed as ACC metastases. The patient is currently on mitotane with no relevant adverse effects (Fig. 2).

This case illustrates the potential for prolonged disease control in metastatic ACC with initially moderate proliferative index, mostly pulmonary disease, through multimodal management integrating systemic therapy (chemotherapy, immunotherapy, mitotane) and repeated local treatments on progressing lesions.

## DISCUSSION

The management of oligometastatic ACC remains a challenging and evolving field. While systemic therapy with mitotane or EDP-M chemotherapy remains the cornerstone for advanced disease, accumulating evidence supports the integration of local treatments in selected patients. Oligometastatic ACC, generally characterized by limited metastatic burden and an indolent biological behavior, such as stage IVa and Ki-67 lower than 20%, may represent a distinct clinical subset where aggressive multimodal strategies can lead to meaningful disease control and prolonged survival.

Retrospective studies and small series suggest that surgery, image-guided thermal ablation (IGT), stereotactic body radiotherapy (SBRT), and transarterial embolization (TACE/TARE) may provide durable local control, especially in patients with ≤5 metastases or lesions smaller than 3 cm. The use of these approaches can postpone systemic chemotherapy, minimize cumulative toxicity, and preserve quality of life. Roux *et al.* and Veltri *et al.* demonstrated that locoregional therapies can achieve disease control rates exceeding 70%, particularly in patients with slow-growing tumors and limited progression. Similarly, combined strategies including mitotane and local interventions have shown median overall survivals up to 68 months in selected cohorts. It should be emphasized that the currently available evidence largely derives from retrospective series and single-institution cohorts. This represents an intrinsic limitation due to heterogeneous inclusion criteria and the lack of prospective randomized data. Moreover, a crucial point beyond the biological and clinical heterogeneity of the tumors themselves is the variable expertise and technological availability among centers performing local therapies. The outcomes reported across studies may therefore reflect not only patient- or disease-related differences but also institutional experience in specific interventional techniques.

Among the various locoregional approaches, thermal ablation (radiofrequency and microwave) represents the most extensively investigated technique, with the largest body of evidence supporting its use. The lung and liver emerge as the most frequently treated metastatic sites, consistent with their predominance in the oligometastatic setting of adrenocortical carcinoma. Although local control rates are encouraging, these findings should be interpreted with caution, as they are influenced by patient selection and procedural expertise.

Decision-making should consider three major domains: patient fitness (age, comorbidities, performance status), tumor biology (Ki-67 index, time to recurrence, metastatic sites), and treatment history (chemosensitivity, prior toxicities, duration of response). Integrating these variables within a multidisciplinary framework enables personalization of therapy and rational sequencing of systemic and local treatments. Immunotherapy has emerged as an option for a minority of patients, showing partial and durable responses in selected cases.

Overall, oligometastatic ACC highlights the potential of a multimodal approach combining systemic control with focused local interventions. Although prospective evidence is lacking, data available are quite heterogenous, multidisciplinary evaluation in a referral center and individualized planning remain essential to optimize survival and quality of life in this rare but clinically significant subset of ACC.

## CONCLUSION

A reasonable definition for ACC oligometastatic disease would include stage IVa or up to five metastases smaller than 3 centimeters, its management required a multidisciplinary approach in referral center in order to choose the best technique and timing for local therapies to be combined with systemic approaches taking into account patient fitness, biology and treatment history.

## Acknowledgements

*AIRC (Associazione Italiana per la Ricerca contro il Cancro), IG23009 (PI: A.B.), IG27233 (PI: S.S.).*

*The authors gratefully acknowledge CreativeLab ASD, school of dance, Livorno, Italy,https://www.facebook.com/creativelabasd in memory of Serena Mazzoni*

*Fondazione Internazionale di Ricerca in Medicina (F.I.R.M.) ONLUS, Cremona (Italy).*

*Mrs Serena Ambrogini and family in memory of her son, Guido Cioni.*

### Financial support and sponsorship

*None.*

### Conflicts of interest

*There are no conflicts of interest.*

## REFERENCES AND RECOMMENDED READING

Papers of particular interest, published within the annual period of review, have been highlighted as:

▪ of special interest
▪▪ of outstanding interest
